# A role for central carbon metabolism in mammalian embryonic development?

**DOI:** 10.1186/2049-3002-2-S1-P69

**Published:** 2014-05-28

**Authors:** Marteinn Snaebjornsson, Nicole Prior, Vinay Bulusu, Bernd Simon, Teresa Carlomagno, Alexander Aulehla

**Affiliations:** 1 Developmental Biology unit, EMBL, Heidelberg, Germany; 2 Structural and Computational Biology unit, EMBL, Heidelberg, Germany

## Background

Embryonic development is not regulated solely by genetic programs but also by metabolic state and environmental cues, which can impact on gene function. Metabolism can influence gene function both canonically (providing substrates for posttranslational modifications etc.) and non-canonically for example via moonlighting enzymes. Moonlighting enzymes have functions in addition to their known catalytic one. They are widespread among enzymes involved in sugar metabolism. For example, 7 out of the 10 enzymes in glycolysis have confirmed roles as moonlighting enzymes [1]. We study the role of glycolysis during mouse embryonic development. To this end, we perturb carbon metabolism in presomitic mesoderm (PSM) explants in culture. Several attributes of the PSM make it an attractive choice for our studies, first of all it can be cultured *in vitro* for extended time periods and displays normal development during culture. This allows us to analyze the effect of our perturbations in a developmental context at the morphological and molecular level.

## Materials and methods

We culture PSM explants in a defined medium in which we can control precisely the nutrient source for the explants, for example replace glucose with a glycolytic intermediate. To analyze the effects of our perturbations we use a transgenic reporter line and real time imaging. We measure lactate secretion and use C13 isotopic labeling to estimate whether and how much glycolytic intermediates are taken up by explants. To search for indications of moonlighting activity of glycolytic enzymes we analyze their localization using subcellular fractionation.

## Results

We find that culture in medium containing F1.6bP more than any other glycoytic metabolite strongly affects PSM development. Additionally we find that a subset of glycolytic enzymes show an unexpected subcellular localization in mouse embryos. Interestingly this localization is altered in the presence of glycolytic intermediates in the culture medium.

## Conclusions

We have evidence suggesting a non-canonical role for glycolysis during embryonic development. Our finding that the localization of glycolytic enzymes can be altered by the presence of metabolites indicates a possible link between metabolic flux and other cellular processes.

## References

[B1] Sriram G Single-gene disorders: what role could moonlighting enzymes play? Am J Hum Genet 2005 76 911 924 10.1086/430799 15877277 PMC1196451

